# Liver transplantation for locally advanced non-resectable intrahepatic cholangiocarcinoma treated with neoadjuvant therapy: early results from the TESLA trial

**DOI:** 10.1093/bjs/znaf054

**Published:** 2025-03-18

**Authors:** Sheraz Yaqub, Sondre Busund, Tor Magnus Smedman, Trygve Syversveen, Ammar Khan, Jon Magnus Solheim, Trine Folseraas, Kristine Wiencke, Kristoffer Lassen, Svein Dueland, Pål-Dag Line

**Affiliations:** Section of Hepato-Pancreato-Biliary (HPB) Surgery, Department of Gastrointestinal and Paediatric Surgery, Oslo University Hospital, Oslo, Norway; Institute of Clinical Medicine, University of Oslo, Oslo, Norway; Section of Hepato-Pancreato-Biliary (HPB) Surgery, Department of Gastrointestinal and Paediatric Surgery, Oslo University Hospital, Oslo, Norway; Institute of Clinical Medicine, University of Oslo, Oslo, Norway; Department of Oncology, Oslo University Hospital, Oslo, Norway; Transplant Oncology Research Group, Division of Surgery and Specialized Medicine, Oslo University Hospital, Oslo, Norway; Department of Radiology and Nuclear Medicine, Oslo University Hospital, Oslo, Norway; Institute of Clinical Medicine, University of Oslo, Oslo, Norway; Transplant Oncology Research Group, Division of Surgery and Specialized Medicine, Oslo University Hospital, Oslo, Norway; Section for Transplant Surgery, Department of Transplantation Medicine, Oslo University Hospital, Oslo, Norway; Institute of Clinical Medicine, University of Oslo, Oslo, Norway; Transplant Oncology Research Group, Division of Surgery and Specialized Medicine, Oslo University Hospital, Oslo, Norway; Section for Transplant Surgery, Department of Transplantation Medicine, Oslo University Hospital, Oslo, Norway; Institute of Clinical Medicine, University of Oslo, Oslo, Norway; Section of Gastroenterology, Department of Transplantation Medicine, Oslo University Hospital, Oslo, Norway; Norwegian PSC Research Centre, Department of Transplantation Medicine, Oslo University Hospital, Oslo, Norway; Section of Gastroenterology, Department of Transplantation Medicine, Oslo University Hospital, Oslo, Norway; Norwegian PSC Research Centre, Department of Transplantation Medicine, Oslo University Hospital, Oslo, Norway; Section of Hepato-Pancreato-Biliary (HPB) Surgery, Department of Gastrointestinal and Paediatric Surgery, Oslo University Hospital, Oslo, Norway; Institute of Clinical Medicine, the Arctic University of Norway, Tromsø, Norway; Transplant Oncology Research Group, Division of Surgery and Specialized Medicine, Oslo University Hospital, Oslo, Norway; Institute of Clinical Medicine, University of Oslo, Oslo, Norway; Transplant Oncology Research Group, Division of Surgery and Specialized Medicine, Oslo University Hospital, Oslo, Norway; Section for Transplant Surgery, Department of Transplantation Medicine, Oslo University Hospital, Oslo, Norway

Intrahepatic cholangiocarcinoma (iCCA) is an aggressive biliary tract cancer with high mortality rate and the incidence is increasing. Surgical resection is the only widely accepted curative option, but up to 80% of patients are unresectable at diagnosis, making systemic therapy the first-line treatment^1^. Long-term survival remains poor, with under 10% surviving beyond 5 years. Liver transplantation has emerged as a potential alternative option for selected patients with non-resectable iCCA, expanding the boundaries of conventional liver resection^2–4^. The TESLA trial evaluates outcomes in patients after liver transplantation for locally advanced, non-resectable, liver-confined iCCA with prior response to neoadjuvant therapy (*Fig. S1*). Here, we report short-term outcomes of the first TESLA patients.

 Methodology and patient selection details are presented in the Supplementary material. Between 8 July 2020 and 31 December 2024, six patients were enrolled in the TESLA study and five underwent liver transplantation after neoadjuvant therapy. Patient and tumour characteristics of the transplanted are presented in *Table 1* and *Table S1*, and pretransplant images in *Fig. 1*. The median age at diagnosis was 45 years, with 60% female. The median number of tumours was 7 (i.q.r. 1–20) and the median diameter 11.5 cm. All patients received at least one gemcitabine-based chemotherapy regimen (*Table S2*). One patient with severe primary sclerosing cholangitis with cirrhosis did not tolerate gemcitabine–cisplatin, but successfully underwent liver transplantation after selective internal radiation therapy with yttrium-90. Next-generation sequencing (NGS) analysis was performed in all patients at time of diagnosis. Only two patients were found to have mutations; *ARID1A* and *KRAS* mutations in one patient and *FGFR2* rearrangement in the other (*Table S3*).

**Fig. 1:**
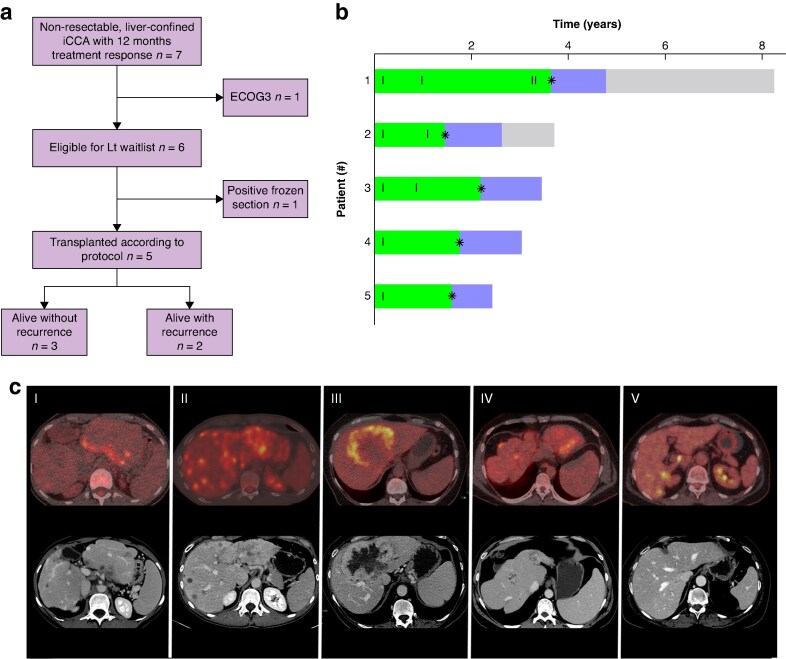
Overview of patients with intrahepatic cholangiocarcinoma (iCCA) undergoing liver transplantation **a** Flowchart of eligible patients in the TESLA trial. During the study period (June 2020—December 2024) patients from all of Norway with non-resectable, liver-confined iCCA were referred for inclusion in the study (*n* = 7). One patient had low performance status (ECOG3) and was found not eligible, another patient was diagnosed with lymph node and peritoneal metastases at diagnostic laparotomy and was not transplanted. A total of 5 patients were transplanted. **b** Swimmer plot illustrates the treatment trajectories of patients with iCCA undergoing liver transplantation. The *x*-axis shows time in years, with 0 as the reference point indicating the time of iCCA diagnosis. Each bar on the *y*-axis corresponds to a case. The green bar indicates the time from diagnosis to liver transplantation (LT), marked by a star (*), whereas the blue segment represents recurrence-free survival following LT, and the grey segment denotes the period following recurrence. Vertical lines indicate neoadjuvant chemotherapy, given in various regimens detailed in Table S3. All patients are alive. **c** Pretransplant images (contrast-enhanced CT and FDG-PET CT) of patients with iCCA undergoing liver transplantation. Panel I: An 11.5 cm tumour involved liver segments VII and VII; some of the multifocal (>20 lesions) bilobar lesions are shown (recipient 1). Panel II: A 13 cm tumour in left liver lobe with multiple (>20 lesions) smaller metastatic lesions involving all liver segments are shown (recipient 2). Panel III: A 12 cm tumour spanning liver segment I, II, IV, V, and VII is shown (recipient 3). Panel IV: A deformed cirrhotic liver due to primary sclerosing cholangitis is shown with single 2.1 cm tumour in right hemi-liver (recipient 4). Panel V: Some of the seven small intrahepatic tumours are shown with the largest being 2 cm (recipient 5).

**Table 1:** Characteristics of patients undergoing liver transplantation for intrahepatic cholangiocarcinoma

*Recipient*	*n = 5*	*1*	*2*	*3*	*4*	*5*
**Demographics**						
Age (years)	45 (32.0–58.0)	28	32	62	45	58
Sex	40% M	F	M	F	M	F
	60% F					
CA19–9 (kU/l, highest value)	198 (43.0–472.0)	490	198	472	43	20
ECOG at transplant	0 (0–0)	0	0	1	0	0
Diagnosis to LT (months)	26 (21,0–26,0)	42	18	26	21	26
Time on waitlist (days)	187 (176.0–193.0)	12	187	214	193	176
**Post-transplant characteristics**						
Follow-up (months)	15 (14.8–26.6)	55	27	15	15	10
Days in hospital	26 (21.0–30.0)	26	19	21	30	30
Postoperative morbidity rate	20%	No	Yes*	No	No	No
**Explant (pathology)**						
Stage	Stage II (T2N0M0)	Stage II (T2N0M0)	Stage IVa (T4N0M0)	Stage II (T2N0M0)	Stage I (T1aN0M0)	Stage IVa (T2N1M0)
Number of lesions	2 (2–20)	>20	>20	2	1	2
Size largest lesion (cm)	5.5 (5.0–8.5)	5.5	8.5	12.2	5.0	1.1
Location	NA	Bilobar	Bilobar	Bilobar	Right	Right
Differentiation	Moderate	NA	Poor	Moderate	NA	Well
Lymph nodes (metastatic/total)	0/6 (6–8)	0/8	0/6	0/8	0/6	1/6
**Patient outcomes**						
Post-transplant recurrence	2 (40%)	Yes	Yes	No	No	No
Time of recurrence (months)	12.5 (12.3–12.8)	13	12	NA	NA	NA
Duration of follow-up post-transplant (months)	15 (14.8–26.6)	55	27	15	15	10

Data are median (i.q.r.) or *n* (%) unless otherwise indicated. ECOG, Eastern Cooperative Oncology Group; LT, liver transplant; NA, not appropriate. *Acute hepatic artery thrombosis leading to re-transplantation of liver.

All liver transplantations were performed at the national transplant centre at OUH. One patient required re-transplantation due to hepatic artery thrombosis. The median length of hospital stay was 26 days (range 19–30). None of the five patients had lymph node metastases on frozen sections. Final pathology confirmed mass-forming iCCA in all patients. However, one patient had malignant cells in a 2 mm lymph node, staged as T2N1M0. No patient received post-transplant adjuvant chemotherapy. Follow-up time from liver transplantation ranges from 10 to 55 months. Two patients developed disease recurrence, presenting with lymph node metastases and lung metastases at 12 and 13 months post-transplantation respectively (*Fig. 1* and *Fig. S2*). All five patients remain alive (*Table 1*).

Tumour size and number are known risk factors for recurrence following liver resection for iCCA^5^. However, tumour pathology and absence of response to neoadjuvant therapy are stronger predictors than tumour size. In our study, patient selection was based on disease confinement to the liver on preoperative imaging and radiological response to neoadjuvant therapy, rather than tumour size or number. However, early recurrence in two patients with large, multifocal tumours suggests multifocal iCCA may not be an optimal indication for liver transplantation. A recent study suggests that combining systemic chemotherapy with SIRT can improve outcome^4^. Future research may explore whether SIRT prior to durvalumab–gemcitabine–cisplatin regimen could enhance the antitumour immune response and improve transplant outcomes. Hepatic artery infusion pump chemotherapy with floxuridine shows promise for non-resectable iCCA and may identify patients with liver-confined, non-metastatic disease who would benefit from liver transplantation.

Advances in NGS have greatly enhanced the understanding of genetic mutations associated with iCCA^1^. In the current series, one patient with both *KRAS* and *ARID1A* mutations, along with multifocal disease, experienced recurrence 13 months post-transplant. Conversely, another patient with an *FGFR2* rearrangement, who responded to erdafitinib prior to liver transplantation, remains disease-free 15 months after transplantation.

The current findings support the notion that liver transplantation in carefully selected patients with locally advanced, liver-limited iCCA might improve survival. Further research on genetic profiling and combining liver transplantation with systemic and locoregional therapies may further improve patient selection and outcomes of liver transplantation for iCCA.

## Supplementary Material

znaf054_Supplementary_Data

## Data Availability

Owing to restrictions on participant consent and data-sharing policies, the supporting data are not publicly available.
