# Supplementary material for: Liver transplantation for locally advanced non-resectable intrahepatic cholangiocarcinoma treated with neoadjuvant therapy: early results from the TESLA trial
Source: Br J Surg. 2025 Mar 18;112(3):znaf054. doi: 10.1093/bjs/znaf054 (PMC11914714; doi:10.1093/bjs/znaf054)
Supplement: znaf054_Supplementary_Data [file znaf054_supplementary_data.docx]

**Liver transplantation for locally advanced non-resectable intrahepatic cholangiocarcinoma treated with neoadjuvant therapy: early results from the TESLA trial**

Sheraz Yaqub^1,2^, Sondre Busund^1,2,*^, Tor Magnus Smedman^3,4,*^, Trygve Syversveen^5^, Ammar Khan^6,2,4^, Jon Magnus Solheim^6,2,4^, Trine Folseraas^7,8,2^, Kristine Wiencke^7,8^, Kristoffer Lassen^1,9^, Svein Dueland^4^, Pål-Dag Line^6,2,4^

1. Section of Hepato-pancreato-biliary (HPB) Surgery, Department of Gastrointestinal and Paediatric Surgery, Oslo University Hospital, Oslo, Norway.
2. Institute of Clinical Medicine, University of Oslo, Oslo, Norway.
3. Department of Oncology, Oslo University Hospital, Oslo, Norway.
4. Transplant Oncology Research Group, Division of Surgery and Specialized Medicine, Oslo University Hospital, Oslo, Norway.
5. Department of Radiology and Nuclear Medicine, Oslo University Hospital, Oslo, Norway.
6. Section for Transplant Surgery, Department of Transplantation Medicine, Oslo University Hospital, Oslo, Norway.
7. Section of Gastroenterology, Department of Transplantation Medicine, Oslo University Hospital, Oslo, Norway.
8. Norwegian PSC Research Centre, Department of Transplantation Medicine, Oslo University Hospital, Oslo, Norway.
9. Institute of Clinical Medicine, the Arctic University of Norway, Tromsø, Norway.

*Both authors contributed equally to this work.

**Corresponding author:**

Sheraz Yaqub, MD PhD FEBS

Department of Hepatopancreatobiliary Surgery

Oslo University Hospital

Oslo N-0372

Norway

E-mail: [sheraz.yaqub@medisin.uio.no](mailto:sheraz.yaqub@medisin.uio.no)

Twitter/X: @yaqubsheraz

ORCID: <https://orcid.org/0000-0002-5696-2319>

**Supplementary Material - Index**

| **Supplementary Methods** | *page 3* |
| --- | --- |
| Study design and participants | *page 3* |
| Neoadjuvant oncological treatment | *page 3* |
| Liver transplantation | *page 3* |
| Follow-up and outcomes | *page 4* |
| Statistical analysis | *page 4* |
| Ethical consideration | *Page 4* |
| **Supplemental Appendixes** | *page 5* |
| Discussion and Limitations | *page 5* |
| Conclusion | *page 6* |
| **Supplementary Figures and Tables** | *page 7* |
| Figure S1 | *page 7* |
| Figure S2 | *Page 8* |
| Table S1 | *Page 9* |
| Table S2 | *page 10* |
| Table S3 | *page 10* |
| **References** | *page 11* |

**Supplementary Methods**

*Study design and participants*

This case series reports the outcomes of the TESLA trial, initiated in 2020, investigating liver transplantation (LT) for patients with locally advanced, non-resectable intrahepatic cholangiocarcinoma (iCCA). Locally advanced iCCA was defined as a solitary or multifocal tumour confined to the liver, with no restrictions on tumour size or number, and without radiological evidence of extrahepatic spread (based on contrast-enhanced computed tomography (CT) and fluorodeoxyglucose (FDG)-positron emission tomography (PET) scans), macrovascular invasion, or lymph node involvement. Unresectability could also result from underlying liver disease. The inclusion and exclusion criteria are shown in Figure S1.

Eligibility for the TESLA study required a minimum of 12 months of disease stability and tumour regression following neoadjuvant therapy. Selection of potential candidates was done by a multidisciplinary tumour board at Oslo University Hospital (OUH), compromising medical oncologists, hepatobiliary and transplant surgeons, hepatologists, interventional radiologists, and diagnostic liver radiologists. Patients deemed suitable underwent a comprehensive screening interview and a detailed medical evaluation by a hepatologist and transplant surgeon. Those meeting the criteria were referred for formal LT evaluation and listing. During the study period, seven patients with liver-confined iCCA who responded to chemotherapy 12 months after diagnosis were referred to this nationwide study. One patient was excluded due to ECOG performance status of 3. Of the six listed patients, one was found to have carcinomatosis and lymph node metastases during staging laparotomy, was not transplanted, and was therefore excluded from this report (Figure 1A).

Histological confirmation of iCCA via biopsy was mandatory for study inclusion. A multidisciplinary team assessed tumour resectability, considering tumour extent and underlying liver disease, with final consensus determined by hepatobiliary and transplant surgeons at OUH. Prior surgical resection for iCCA was permitted if performed at least 12 months before listing, with pathology confirming R0N0 status. The same duration of disease response was required for both previously resected and non-resected patients.

*Neoadjuvant oncological treatment*

Neoadjuvant therapy primarily consisted of gemcitabine-based regimens, such as gemcitabine-cisplatin or gemcitabine-capecitabine, with gemcitabine-cisplatin administered as standard of care per the ABC-02 protocol^1^. If patients were intolerant to first-line therapy, the regimen that achieved disease control was continued until transplantation. In cases of intrahepatic disease progression, next-line therapies including fluoropyrimidines or targeted agents for actionable mutations identified via next-generation sequencing (NGS)^2^, were administrated per institutional guidelines (Table S2). Durvalumab^3^ was not approved for standard iCCA treatment in Norway before June 2024. All patients underwent CT scans and tumour marker assessments after four chemotherapy cycles, followed by imaging every two months. Disease stability was reassessed bi-monthly while on the waitlist. Patients with extrahepatic progression were not included in the study and continued with palliative chemotherapy.

*Liver transplantation*

Patients were only listed for LT if they demonstrated at least a 10% tumour response and liver-limited disease based on FDG-PET CT scan. LT was performed using extended-criteria donors (ECD) liver grafts that would otherwise not be used for transplantation. These included Donation after Circulatory Death (DCD) livers, donors with a history of low-to-moderate risk malignancy (per SaBTO criteria), and those with treatable viral hepatitis but normal liver function.

Criteria for marginal grafts:

- DCD livers: Higher risk of ischemic injury but accepted in cases with high urgency.
- Transferable diseases:
- HBV+ donors: Low transmission risk with proper prophylaxis.
- HCV+ donors: Universally transmitted but treatable with direct-acting antivirals.
- Malignancy: Donors classified as low-to-intermediate risk per international guidelines.
- Other factors: Moderate steatosis (>30%), older donors (>80 years), and certain metabolic diseases.

These criteria allowed for the inclusion of ECD liver grafts for patients with malignant liver tumours. If there was no matching recipient for a liver graft, some patients received standard donor grafts. No organs underwent machine perfusion before transplantation. At the start of LT, a staging laparotomy with lymph nodes sampling from the hepatoduodenal ligament was performed. If frozen-section analysis confirmed the presence of extrahepatic or nodal metastases, the surgery was aborted. All LTs were performed at the national transplant centre at OUH.

*Follow-up and outcomes*

Postoperative adjuvant chemotherapy was not administered. Patients underwent intensive post-transplant surveillance for disease recurrence, including CA19-9 monitoring and CT or MRI scans every three months for two years, then biannually for up to five years. Beyond five years, the protocol describes imaging performed annually. Recurrences were treated aggressively with surgery, radiation, or chemotherapy as indicated. Patient data were collected prospectively and longitudinally from the time of study inclusion, while data from the time of diagnosis was retrospectively obtained from the referring hospitals. The primary endpoint was survival, and secondary endpoints included disease-free survival, Quality of life (assessed by EORTC QLQ-C30), and postoperative complications graded using the Clavien-Dindo classification (grades 3-5)^4^.

*Ethical consideration*

The TESLA protocol was approved by the Regional Committees for Medical and Health Research Ethics (REK 96545) and Data Protection Officer at OUH (20/06063). The study is registered in Clinicaltrials.gov #: NCT04556214. All patients provided written informed consent before listing, which occurred between June 26, 2020, and October 26, 2023.

*Statistical Analysis*

For this analysis, all data were censored as of December 31, 2024. Descriptive statistics were calculated for the cohort. Categorical variables were presented as numbers (percentages), while numerical variables as median (interquartile range). All analyses were performed using IBM SPSS Statistics for Windows, version 26 (IBM Corp., United States).

**Supplementary Appendixes**

*Discussion and Limitations*

In this cohort of patients undergoing LT for non-resectable iCCA following downstaging with neoadjuvant therapy, we report that three patients show no evidence of disease. Identifying patients with liver-confined iCCA who respond to tumour-directed therapy, such as systemic chemotherapy^3^, targeted therapy^5^, or local treatment with selective internal radiation therapy (SIRT)^6^ or hepatic artery infusion pump (HAIP)^7^, helps define a subgroup eligible for LT, offering a potential opportunity to overcome the dismal prognosis of non-resectable iCCA.

Tumour size is a well-established risk factor for recurrence following liver resection for iCCA^8^. Several retrospective studies have suggested a potential link between tumour size and post-transplant recurrence^9, 10^. One study found that patients with multiple tumours or with tumours larger than 5 cm in diameter had the highest risk of recurrence^11^. However, a separate risk analysis of recurrence after LT for iCCA indicated that tumour pathology and the absence of neoadjuvant therapy were stronger independent predictors of recurrence than tumour size^12^. In our study, we followed a strategy similar to that of the Methodist–MD Anderson group^13^. Patient selection was based on disease confinement to the liver on preoperative imaging and a demonstrated radiological response to neoadjuvant therapy, rather than tumour size or number. However, we observed that the two patients with large tumours and multifocal bilobar disease experienced early recurrence, suggesting that multifocal iCCA may not be an optimal indication for LT.

The introduction of more effective neoadjuvant therapy, particularly the gemcitabine and cisplatin regimen^1^ has renewed interest in LT as a treatment option for non-resectable iCCA. Early reports demonstrated promising outcomes when neoadjuvant treatment was used as a selection tool for LT^13, 14^. A recent study suggests that combining systemic chemotherapy with radioembolization using SIRT can further enhance treatment effectiveness^15^. In patients with liver-confined, node negative, and non-resectable iCCA, this approach led to over 70% tumour response and significantly improved survival outcomes following transplantation. Additionally, advancements in systemic therapy, such as incorporating durvalumab to gemcitabine-cisplatin regimen^3^, have yet to be evaluated in the LT setting. Future research may also explore whether adding SIRT could trigger an abscopal effect, enhancing the antitumor immune response and potentially improving transplant outcomes^16^.

Tumour biology likely played a significant role in the outcomes of our patients, as the selected patients demonstrated sustained responses to chemotherapy. Advances in NGS and the widespread availability of genetic profiling have greatly enhanced the understanding of genetic mutations associated with iCCA^2, 5^. Certain mutations, such as AT-rich interaction domain 1A (*ARID1A*) and *KRAS*^17, 18^, have been linked to more aggressive tumour biology, whereas fibroblast growth factor receptor (*FGFR2*) fusions have been associated with a more favourable prognosis. In our series, one patient with both *KRAS* and *ARID1A* mutations (Table S3), along with multifocal disease with more than 20 intrahepatic tumours, experienced recurrence 13 months post-transplant. Conversely, another patient with an *FGFR2* rearrangement, who responded to erdafitinib prior to LT, remains disease-free 15 months after transplantation. None of the five patients treated with LT had a microsatellite instable (MSI) tumour (Table S3). In the future, molecular profiling might help identify patients with targetable mutations and favourable tumour biology who are more likely to benefit from transplantation, as opposed to those with a higher risk of post-transplant recurrence.

Over the past decade, HAIP chemotherapy with floxuridine (FUDR) has shown promising outcomes compared to systemic chemotherapy in patients with non-resectable iCCA^7, 19^. The treatment is particularly appealing due to the high first-pass metabolism of FUDR, with approximately 95% being directly metabolized by the liver. However, a known complication of HAIP therapy is FUDR-induced biliary sclerosis, which in some cases has necessitated salvage LT^20^. Since HAIP primarily targets liver-confined disease, it may serve as tool to identify patients with truly non-metastatic iCCA who could benefit from LT. Nevertheless, it is important to acknowledge that LT following HAIP therapy presents technical challenges, which have been previously documented^20^.

Circulating tumour DNA (ctDNA) has emerged as a promising, non-invasive biomarker for detecting and monitoring solid organ malignancies. However, its role in patients with primary and secondary liver cancers before and after LT remains to be explored. In a recent study, ctDNA positivity was associated with a higher recurrence rate post-transplant, indicating its potential as a predictor of disease relapse following curative-intent LT^21^.

This study has several limitations. It is a single centre experience with a small sample size and its findings are based on a highly selected patient population due to strict inclusion criteria. Additionally, the absence of a control group limits the ability to directly assess the survival benefit of LT. Due to nationwide recruitment and the requirement for referral only after 12 months of chemotherapy response, we lack data on the number of patients who experienced disease progression during this period or those who underwent liver resection instead. Neoadjuvant chemotherapy was administered according to the standard of care at the time i.e., gemcitabine-cisplatin regimen, without addition of durvalumab, which is now the standard for non-resectable iCCA. Lastly, three of the five patients who underwent LT have less than two years of follow-up, limiting long-term outcome assessment.

*Conclusion*

Our findings support the notion that LT in carefully selected patients with locally advanced, liver-limited iCCA might improve survival outcomes. Future research focusing on refining patient selection, genetic profiling, and combining LT with systemic and locoregional therapies may further improve survival outcomes and broaden the applicability of LT for iCCA.

**Supplementary Figures and Tables**

**Figure S1. The TESLA study diagram.** The chart shows the selection criteria for inclusion and exclusion in the study.


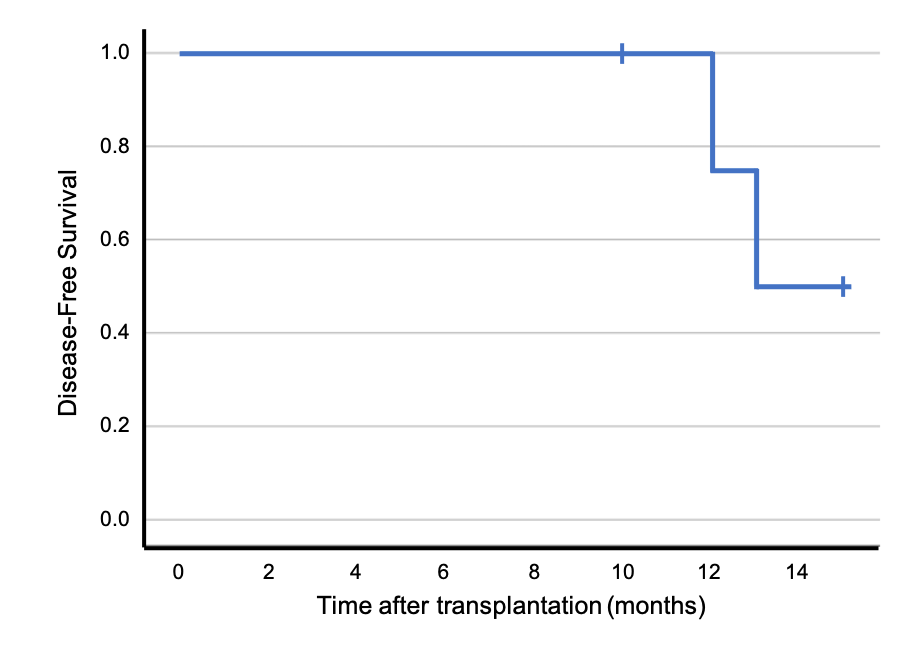


**Figure S2. Disease-Free survival after liver transplantation for intrahepatic cholangiocarcinoma.** Kaplan-Meier curve for five patients transplanted in the TESLA-study.

**Table S1.** Characteristics of patients undergoing liver transplantation for intrahepatic cholangiocarcinoma.

|  | n=5 | Recipient 1 | Recipient 2 | Recipient 3 | Recipient 4 | Recipient 5 |
| --- | --- | --- | --- | --- | --- | --- |
| **Demographics** |  | | | | | |
| Age (years) | 45 (32.0-58.0) | 28 | 32 | 62 | 45 | 58 |
| BMI | 24 (23.2-26.6) | 18.1 | 27.2 | 23.2 | 26.6 | 24.0 |
| Sex | M:F (40/60%) | F | M | F | M | F |
| PSC | 2 (40%) | No | No | No | Yes | Yes |
| CA19-9 (kU/L, highest value) | 198 (43.0-472.0) | 490 | 198 | 472 | 43 | 20 |
| Diabetes | 0 (0%) | No | No | No | No | No |
| Hypertension | 0 (0%) | No | No | No | No | No |
| Smoking | 0 (0%) | No | No | No | No | No |
| ECOG at transplant | 0 (0-0) | 0 | 0 | 1 | 0 | 0 |
| Karnofsky score at transplant | 90 (90.0-100.0) | 100 | 100 | 80 | 90 | 90 |
| Diagnosis to LT (months) | 26 (21,0-26,0) | 42 | 18 | 26 | 21 | 26 |
| Time on waitlist (days) | 187 (176.0-193.0) | 12 | 187 | 214 | 193 | 176 |
| **Operative characteristics** |  | | | | | |
| Venovenous bypass | 4 (80%) | Yes | Yes | Yes | No | Yes |
| Major reperfusion syndrome | 0 (0%) | 0 | 0 | 0 | 0 | 0 |
| Cold ischemia time (min) | 308 (254.0-366.0) | 420 | 308 | 366 | 163 | 254 |
| **Post-transplant characteristics** |  | | | | | |
| Follow-up (months) | 15 (14.8-26.6) | 55 | 27 | 15 | 15 | 10 |
| Days in hospital | 26 (21.0-30.0) | 26 | 19 | 21 | 30 | 30 |
| Death 90 days | 0 | No | No | No | No | No |
| Postoperative morbidity | 20% | No | Yes* | No | No | No |
| **Radiographic (pre-transplant)** |  | | | | | |
| Stage | Stage II (T2bN0M0) | Stage II (T2bN0M0) | Stage II (T2bN0M0) | Stage I (T1N0M0) | Stage I  (T1N0M0) | Stage II (T2bN0M0) |
| Number of lesions | 7 (1-20) | >20 | >20 | 1 | 1 | 7 |
| Size largest lesion (cm) | 11.5 (2.1-12.0) | 11.5 | 13.0 | 12 | 2.1 | 2,0 |
| **Explant (pathology)** |  | | | | | |
| Stage | Stage II (T2N0M0) | Stage II (T2N0M0) | Stage IVa (T4N0M0) | Stage II (T2N0M0) | Stage I  (T1aN0M0) | Stage IVa (T2N1M0) |
| Number of lesions | 2 (2-20) | >20 | >20 | 2 | 1 | 2 |
| Size largest lesion (cm) | 5.5 (5.0-8.5) | 5,5 | 8.5 | 12.2 | 5.0 | 1.1 |
| Location | NA | Bilobar | Bilobar | Bilobar | Right | Right |
| Differentiation | Moderate | NA | Poor | Moderate | NA | Well |
| Lymph nodes (metastatic/total) | 0/6 (6-8) | 0/8 | 0/6 | 0/8 | 0/6 | 1/6 |
| Lymphovascular invasion | No | No | No | No | No | No |
| Perineural invasion | No | No | Yes | No | No | No |
| Microvascular invasion | No | No | No | No | No | No |
| Macrovascular invasion | No | No | No | No | No | No |
| Positive margins | No | No | No | No | No | No |
| **Patient outcomes** |  | | | | | |
| Post-transplant recurrence | 2 (40%) | Yes | Yes | No | No | No |
| Time of recurrence (months) | 12.5 (12.3-12.8) | 13 | 12 | NA | NA | NA |
| Duration of follow-up post-transplant (months) | 15 (14.8-26.6) | 55 | 27 | 15 | 15 | 10 |
| Duration of follow-up from diagnosis (months) | 48.3 (42.2-50.8) | 97 | 51 | 48 | 42 | 41 |

Data are median (interquartile range, IQR) or n (%). BMI=body mass index, PSC=primary sclerosing cholangitis. ECOG= Eastern Cooperative Oncology Group, LT= liver transplant, NA=not appropriate. *Acute hepatic artery thrombosis leading to re-transplantation of liver.

**Table S2.** Details of neoadjuvant therapy in liver transplant recipients with intrahepatic cholangiocarcinoma.

| Patient# | Time from diagnosis to transplant (months) | Status | Neoadjuvant treatment therapies (treatment line) | | | |
| --- | --- | --- | --- | --- | --- | --- |
|  |  |  | 1 | 2 | 3 | 4 |
| **1** | 42 | Alive with metastatic disease | Gemcitabine, cisplatin | Gemcitabine | Gemcitabine, oxaliplatin | Gemcitabine, capecitabine |
| **2** | 18 | Alive with metastatic disease | Gemcitabine, oxaliplatin | Gemcitabine, capecitabine | Fluorouracil, oxaliplatin, irinotecan |  |
| **3** | 26 | Alive without recurrence | Gemcitabine, cisplatin | Fluorouracil, irinotecan | Erdafitinib |  |
| **4** | 21 | Alive without recurrence | Gemcitabine, cisplatin | SIRT |  |  |
| **5** | 26 | Alive without recurrence | Gemcitabine, cisplatin |  |  |  |

SIRT: Selective Internal Radiation Therapy.

**Table S3.** Genetic mutation analysis of liver transplant recipients with intrahepatic cholangiocarcinoma.

|  | Recipient 1 | Recipient 2 | Recipient 3 | Recipient 4 | Recipient 5 |
| --- | --- | --- | --- | --- | --- |
| ARID1A | P1209fs | 0 | 0 | 0 | 0 |
| BRAF | 0 | 0 | 0 | 0 | 0 |
| BRCA 1/2 | 0 | 0 | 0 | 0 | 0 |
| ERBB2 | 0 | 0 | 0 | 0 | 0 |
| FGFR2 | 0 | 0 | Rearrangement | 0 | 0 |
| IDH 1/2 | 0 | 0 | 0 | 0 | 0 |
| KRAS | G12D | 0 | 0 | 0 | 0 |
| MSH6 | 0 | 0 | 0 | 0 | 0 |
| MSI/MSS | MSS | MSS | MSS | MSS | MSS |
| MYC | 0 | 0 | 0 | 0 | 0 |
| NTRK 1/2/3 | 0 | 0 | 0 | 0 | 0 |
| PTEN | 0 | 0 | 0 | 0 | 0 |
| SMAD4 | 0 | 0 | 0 | 0 | 0 |

**References**

1. Valle JW, Wasan H, Johnson P, Jones E, Dixon L, Swindell R, et al. Gemcitabine alone or in combination with cisplatin in patients with advanced or metastatic cholangiocarcinomas or other biliary tract tumours: a multicentre randomised phase II study - The UK ABC-01 Study. *Br J Cancer* 2009;101:621-627

2. Vogel A, Bridgewater J, Edeline J, Kelley RK, Klumpen HJ, Malka D, et al. Biliary tract cancer: ESMO Clinical Practice Guideline for diagnosis, treatment and follow-up. *Ann Oncol* 2023;34:127-140

3. Oh DY, He AR, Bouattour M, Okusaka T, Qin S, Chen LT, et al. Durvalumab or placebo plus gemcitabine and cisplatin in participants with advanced biliary tract cancer (TOPAZ-1): updated overall survival from a randomised phase 3 study. *Lancet Gastroenterol Hepatol* 2024;9:694-704

4. Dindo D, Demartines N, Clavien PA. Classification of surgical complications: a new proposal with evaluation in a cohort of 6336 patients and results of a survey. *Ann Surg* 2004;240:205-213

5. Lamarca A, Barriuso J, McNamara MG, Valle JW. Molecular targeted therapies: Ready for "prime time" in biliary tract cancer. *J Hepatol* 2020;73:170-185

6. Edeline J, Bridgewater J, Campillo-Gimenez B, Neveu E, Phelip JM, Neuzillet C, et al. Chemotherapy with or without selective internal radiation therapy for intrahepatic cholangiocarcinoma: Data from clinical trials. *Hepatology* 2024;79:96-106

7. Holster JJ, El Hassnaoui M, Franssen S, JNM IJ, de Jonge J, Mostert B, et al. Hepatic Arterial Infusion Pump Chemotherapy for Unresectable Intrahepatic Cholangiocarcinoma: A Systematic Review and Meta-Analysis. *Ann Surg Oncol* 2022;29:5528-5538

8. Spolverato G, Kim Y, Ejaz A, Alexandrescu S, Marques H, Aldrighetti L, et al. Conditional Probability of Long-term Survival After Liver Resection for Intrahepatic Cholangiocarcinoma: A Multi-institutional Analysis of 535 Patients. *JAMA Surg* 2015;150:538-545

9. Friman S, Foss A, Isoniemi H, Olausson M, Hockerstedt K, Yamamoto S, et al. Liver transplantation for cholangiocarcinoma: selection is essential for acceptable results. *Scand J Gastroenterol* 2011;46:370-375

10. Sapisochin G, Facciuto M, Rubbia-Brandt L, Marti J, Mehta N, Yao FY, et al. Liver transplantation for "very early" intrahepatic cholangiocarcinoma: International retrospective study supporting a prospective assessment. *Hepatology* 2016;64:1178-1188

11. Zhang XF, Beal EW, Bagante F, Chakedis J, Weiss M, Popescu I, et al. Early versus late recurrence of intrahepatic cholangiocarcinoma after resection with curative intent. *Br J Surg* 2018;105:848-856

12. Hong JC, Petrowsky H, Kaldas FM, Farmer DG, Durazo FA, Finn RS, et al. Predictive index for tumor recurrence after liver transplantation for locally advanced intrahepatic and hilar cholangiocarcinoma. *J Am Coll Surg* 2011;212:514-520; discussion 520-511

13. Lunsford KE, Javle M, Heyne K, Shroff RT, Abdel-Wahab R, Gupta N, et al. Liver transplantation for locally advanced intrahepatic cholangiocarcinoma treated with neoadjuvant therapy: a prospective case-series. *Lancet Gastroenterol Hepatol* 2018;3:337-348

14. McMillan RR, Javle M, Kodali S, Saharia A, Mobley C, Heyne K, et al. Survival following liver transplantation for locally advanced, unresectable intrahepatic cholangiocarcinoma. *Am J Transplant* 2022;22:823-832

15. Maspero M, Sposito C, Bongini MA, Cascella T, Flores M, Maccauro M, et al. Liver Transplantation for Intrahepatic Cholangiocarcinoma After Chemotherapy and Radioembolization: An Intention-To-Treat Study. *Transpl Int* 2024;37:13641

16. Bernstein MB, Krishnan S, Hodge JW, Chang JY. Immunotherapy and stereotactic ablative radiotherapy (ISABR): a curative approach? *Nat Rev Clin Oncol* 2016;13:516-524

17. Zhao S, Xu Y, Wu W, Wang P, Wang Y, Jiang H, et al. ARID1A Variations in Cholangiocarcinoma: Clinical Significances and Molecular Mechanisms. *Front Oncol* 2021;11:693295

18. Zhu AX, Borger DR, Kim Y, Cosgrove D, Ejaz A, Alexandrescu S, et al. Genomic profiling of intrahepatic cholangiocarcinoma: refining prognosis and identifying therapeutic targets. *Ann Surg Oncol* 2014;21:3827-3834

19. Cercek A, Boerner T, Tan BR, Chou JF, Gonen M, Boucher TM, et al. Assessment of Hepatic Arterial Infusion of Floxuridine in Combination With Systemic Gemcitabine and Oxaliplatin in Patients With Unresectable Intrahepatic Cholangiocarcinoma: A Phase 2 Clinical Trial. *JAMA Oncol* 2020;6:60-67

20. Hill AL, Cullinan DR, Ahmed O, Vachharajani N, Scherer MD, Olumba F, et al. Liver Transplantation After Hepatic Artery Infusion Pump Therapy: Single-Center Experience and Technical Considerations. *Ann Surg Oncol* 2023;30:4775-4780

21. Hong H, Wehrle CJ, Zhang M, Fares S, Stitzel H, Garib D, et al. Circulating Tumor DNA Profiling in Liver Transplant for Hepatocellular Carcinoma, Cholangiocarcinoma, and Colorectal Liver Metastases: A Programmatic Proof of Concept. *Cancers (Basel)* 2024;16:
